# Serum SIRT3 levels in epilepsy patients and its association with clinical outcomes and severity: A prospective observational study

**DOI:** 10.1515/med-2024-1011

**Published:** 2024-07-30

**Authors:** Yun Hu, Ting Zhou, Qingye Li

**Affiliations:** Department of Emergency Medicine, People’s Hospital of Dongxihu District, Wuhan, Hubei, 430040, China; Department of Emergency Medicine, Hubei Provincial Hospital of Traditional Chinese Medicine, Wuhan, China; Department of Neurology, People’s Hospital of Dongxihu District, 48 Jinbei 1st Road, Jinghe Street, Dongxihu District, Wuhan, Hubei, 430040, China

**Keywords:** epilepsy, SIRT3, neuroinflammation, refractory, risk factors

## Abstract

**Objective:**

In this prospective observational study, we aimed to investigate the serum levels of sirtuin (SIRT)3 in epilepsy patients and its association with the severity of the disease.

**Methods:**

This prospective observational study included 203 patients with symptomatic epilepsy and 100 healthy controls who visited our hospital from November 2019 to November 2022. The severity of the disease in epilepsy patients was assessed using the National Hospital Seizure Severity Scale (NHS3). We used enzyme-linked immunosorbent assay to measure the serum levels of SIRT3, interleukin (IL)-6, IL-1β, tumor necrosis factor-alpha, and C-reactive protein in all patients. In addition, the cognitive function of all study participants was evaluated using the Mini-Mental State Examination and the Montreal Cognitive Assessment (MOCA). All data were analyzed using SPSS 25.0 software.

**Results:**

The MOCA scores of the epilepsy patients were significantly lower compared to the healthy volunteers (*P* < 0.05). The serum SIRT3 levels were decreased significantly in patients with refractory epilepsy (183.16 ± 17.22 pg/mL) compared to non-refractory epilepsy patients (199.00 ± 18.68 pg/mL). In addition, serum SIRT3 levels were negatively correlated with the inflammatory factors IL-6 (Pearson’s correlation −0.221, *P* = 0.002) and NHS score (Pearson’s correlation −0.272, *P* < 0.001) of epilepsy patients, while positively correlated with MOCA scores (Pearson’s correlation 0.166, *P* = 0.018). Furthermore, the receiver operating characteristic curve demonstrated that serum SIRT3 could be used to diagnose epilepsy, as well as refractory epilepsy. Finally, logistic regression analysis showed that SIRT3 (OR = 1.028, 95%CI: 1.003–1.054, *P* = 0.028), IL-6 (OR = 0.666, 95%CI: 0.554–0.800, *P* < 0.001), IL-1β (OR = 0.750, 95%CI: 0.630–0.894, *P* = 0.001), and NHS3 (OR = 0.555, 95%CI: 0.435–0.706, *P* < 0.001) were risk factors for refractory epilepsy.

**Conclusion:**

In conclusion, our findings demonstrated that serum SIRT3 levels were significantly decreased in epilepsy patients and further decreased in patients with refractory epilepsy. This study might provide new therapeutic targets and comprehensive treatment strategies for epilepsy patients.

## 1 Introduction

Epilepsy is one of the most common neurological disorders, affecting over 70 million people worldwide [1]. It is characterized by recurrent seizures, including convulsions, muscle spasms, rigidity, brief loss of consciousness, tonic episodes, prolonged muscle contractions, and absence seizures [2]. The prevalence of epilepsy is estimated to be 1–2%, and it can occur in individuals of all ages, leading to significant physiological, psychological, social, and economic burdens for patients, caregivers, and society [3]. Therefore, improving the prognosis of epilepsy patients and controlling seizure activity is of paramount importance.

The pathogenesis of epilepsy is associated with various mechanisms that lead to abnormal neuronal activity, including oxidative stress, excitotoxicity mediated by glutamate, mitochondrial dysfunction, and high levels of inflammation [4,5]. There is evidence to suggest that inflammation may be both a consequence and a cause of epilepsy, as various inflammatory mediators have been detected in brain tissues resected from patients with refractory epilepsy [6]. In recent years, the sirtuin (SIRT) family has been reported to play diverse roles in different inflammatory diseases [7]. Among them, SIRT3, a NAD± dependent deacetylase primarily located in mitochondria, functions to maintain mitochondrial homeostasis under stress conditions [8]. Increasing evidence has shown that SIRT3 plays significant physiological and pathological roles in neurological disorders [9,10]. Additionally, abnormal expression of the SIRT family has been found in the cerebrospinal fluid of pediatric patients with epilepsy [11]. Furthermore, activation of SIRT3 has been shown to alleviate pathological damage in the hippocampus of epilepsy rat models, promote mitochondrial autophagy, and reduce oxidative stress [12]. In our previous study, we also observed aberrant expression of SITR3 in epilepsy patients (data not published). However, there is currently a lack of research on the expression of SIRT3 in epilepsy patients and its clinical implications, particularly in relation to the severity of the disease.

Therefore, in this prospective observational study, we aimed to investigate the serum levels of SIRT3 in epilepsy patients and its association with the severity of the disease. This research may provide new clinical evidence for the treatment of patients with epilepsy.

## 2 Methods

### 2.1 Participants

This prospective observational study included 203 patients with symptomatic epilepsy who visited our hospital from November 2019 to November 2022. Epilepsy was diagnosed according to the 2006 International League Against Epilepsy Classification by at least two licensed and experienced neurologists [13]. All patients were over 18 years old, had a disease duration of over 3 months, and were diagnosed with primary epilepsy. The exclusion criteria were as follows: (1) secondary epilepsy was excluded based on medical history, neurological examination, and cranial MRI; (2) autoimmune diseases such as rheumatoid arthritis, lupus erythematosus, and dermatomyositis; (3) severe infections, liver or kidney dysfunction, and malignancies; (4) diabetes; and (5) other neurological disorders such as dementia, Parkinson’s disease, and Alzheimer’s disease (AD). In addition, 195 healthy volunteers who came to our hospital for physical examinations were included as controls. All healthy volunteers were matched to the epilepsy patients in terms of age, gender, and BMI. None of them had any underlying organic cardiac or pulmonary conditions. Moreover, their liver and kidney function, blood glucose levels, and chest X-ray results were within the normal range.

### 2.2 Sample size

\[({{\mathrm{Z}}}_{1-\alpha /2}\times \sqrt{p\left\times (1\left-p)}2\left/{\delta }^{2})]\] was used to calculate the sample size. *Z*_1−*α*/2_ = 1.96, *δ* = 0.05, and *p* represents the sensitivity, which in this study is expected to be 85%. Thus, both groups had a minimum sample size of 195 participants.

### 2.3 Blood sampling and analysis

About 5 mL of blood sample after 8 h of fasting were collected from all study participants in the morning (8 h) and were analyzed using enzyme-linked immunosorbent assay (ELISA) to measure the levels of serum SIRT3, interleukin (IL)-6, IL-1β, tumor necrosis factor-alpha (TNF-α), and C-reactive protein (CRP) in all patients. The serum samples were centrifuged at 2,000*g* for 15 min, and the levels of SIRT3, IL-6, IL-1β, TNF-α, and CRP were measured using commercially available ELISA kits (SIRT3 MBS3803577, IL-6 MBS2019894, IL-1β MBS3803011, TNF-α MBS824943, CRP MBS8123937, MyBioSource, California, USA). In brief, ELISA plates were incubated overnight at 4°C with 100 μL of the target protein solution, washed with PBS buffer, and blocked with 1% bovine serum albumin in PBS for 1 h. After further washing with PBS containing 0.05% Tween-20, the bound antibodies were detected by sequential dilutions of the target (culture supernatant, antibody) and incubation at room temperature. After additional washing, binding antibodies were detected using sheep anti-mouse IgG conjugated to HRP. About 100 mL of 3,3′,5,5′-tetramethylbenzidine substrate was added, incubated for 10–20 min, and then 50 μL of stop solution (1NH2SO4) was added. The absorbance at 450 nm was measured.

### 2.4 Outcomes

Demographic data and clinical indicators of all patients were collected, including age, sex, BMI, disease duration, age at onset, family history, monthly seizure frequency, and antiepileptic drugs (AEDs). The cognitive function of all study participants was evaluated using the Mini-Mental State Examination (MMSE) and the Montreal Cognitive Assessment (MOCA). The severity of the disease in patients was assessed using the National Hospital Seizure Severity Scale (NHS3) [14]. Patients with refractory epilepsy were defined based on the criteria proposed by Berg et al., which included the use of two or more AEDs for more than 18 months and still experiencing at least one seizure per month [15].

### 2.5 Statistical analysis

Data were analyzed using SPSS 25.0 software (IBM, Armonk, NY, USA). The normal distribution of data was confirmed using the Kolmogorov–Smirnov test. Normally distributed data were expressed as mean ± SD, while non-normally distributed data were expressed as median (range). Between-group comparisons were performed using the Mann–Whitney test or Student’s *t*-test. Proportions were compared using the chi-square test. One-way analysis of variance followed by Tukey’s *post hoc* test was used for comparison among three groups. Pearson’s correlation analysis was performed to assess the correlations between serum inflammatory factors, SIRT3, and clinical characteristics of epilepsy patients. Receiver operating characteristic (ROC) curve analysis was conducted to determine the diagnostic value of serum SIRT3 levels in epilepsy patients and to discriminate patients with refractory epilepsy. Logistic regression analysis was employed to identify risk factors for refractory epilepsy. Differences were considered statistically significant at *P* < 0.05.


**Ethical approval:** This study has obtained ethical approval from the ethics committee of People’s Hospital of Dongxihu District, with the ethical approval number D2019-07-02.
**Informed consent:** All patients voluntarily participated in this study and provided informed consent by signing the informed consent forms.

## 3 Results

### 3.1 Clinical characteristics of all participants

**Figure 1:**
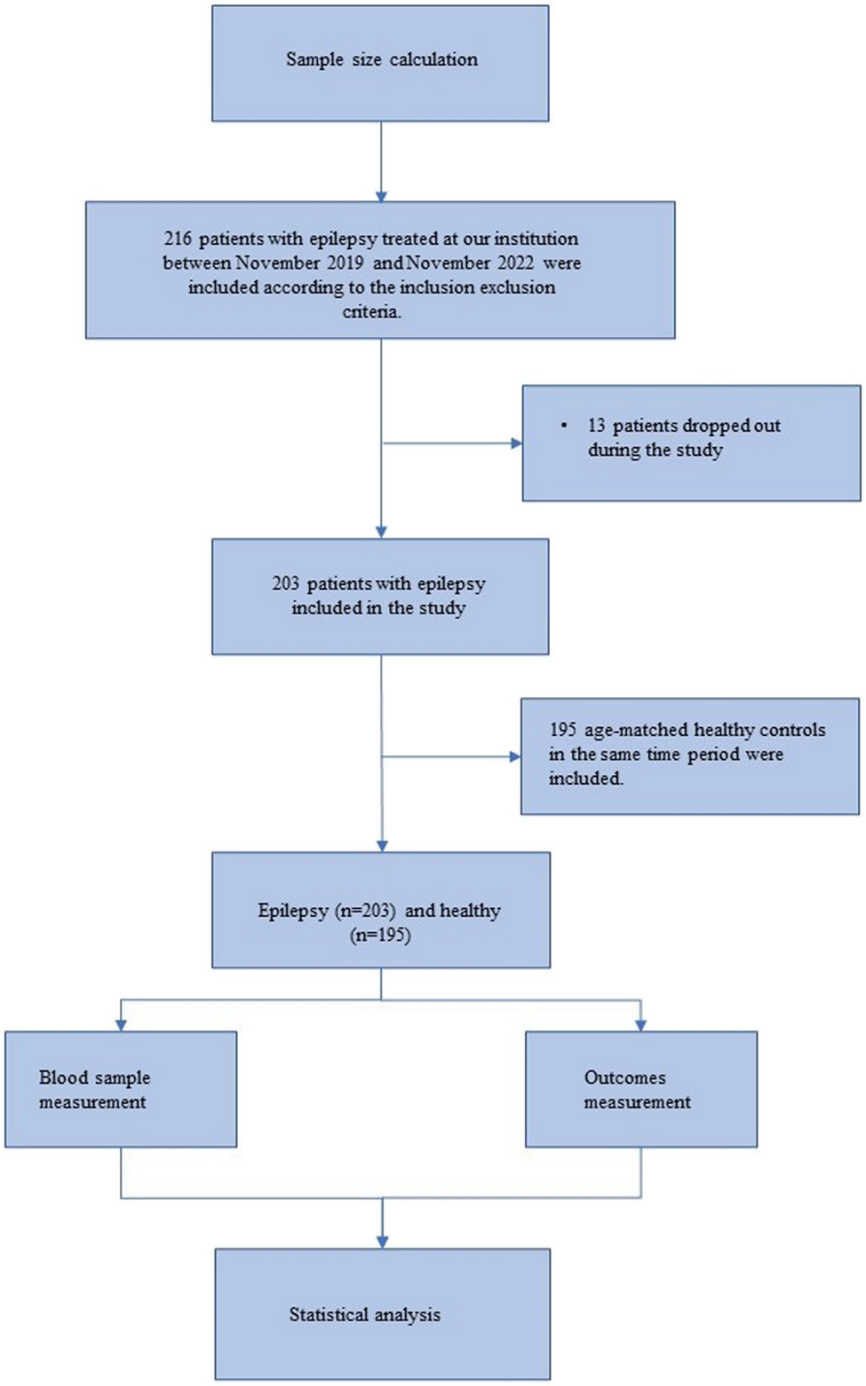
Flow diagram of the research.

The flow diagram is shown in Figure 1, in this prospective observational study, we recruited a total of 203 epileptic patients and 195 healthy controls, with their clinical characteristics shown in Table 1. Among them, there were no significant differences in age, gender, and BMI between epileptic patients and healthy controls. About 43.35% of patients with epilepsy had disease duration greater than 1 year, with an average age of onset at 29.88 ± 6.99 years, and an average NHS score of 10.14 ± 2.35. As shown in the results, we found that the epileptic patients had significantly lower MOCA scores compared to the healthy volunteers (*P* < 0.05).

**Table 1:** Demographic and clinical data of all subjects

Variable	Epilepsy, *n* = 203	Healthy, *n* = 195	*P*
Age, years	31.65 ± 7.39	31.28 ± 7.56	0.623
Sex, female (%)	98 (48.28%)	106 (54.36%)	0.390
BMI	24.79 (19.93–28.83)	24.32 (19.92–28.29)	0.540
MMSE	26.21 ± 1.31	27.27 ± 0.87	0.352
MOCA	24.45 ± 1.80	27.84 ± 0.87	<0.001
Age at onset	29.88 ± 6.99		
**Disease duration**			
<1 year	115 (56.65%)		
≥1 year	88 (43.35%)		
**Monthly seizure frequency**			
<2/month	97 (47.78%)		
≥2/month	106 (52.22%)		
**AEDs**			
<2 types	75 (36.95%)		
≥2 types	128 (63.05%)		
Family history of epilepsy, *n* (%)	7 (3.45%)		
NHS3	10.14 ± 2.35		

BMI: body mass index; MMSE: Mini-Mental State Examination; MOCA: Montreal Cognitive Assessment; NHS3: National Hospital Seizure Severity Scale. Continuous data presented non-normal distribution were expressed by median (minimum to maximum) and analyzed by Mann–Whitney *U*-test. Continuous data presented normal distribution were expressed by mean ± SD and analyzed by Student’s *t*-test. Chi square test was used for rates.

### 3.2 Differential expression of serum biomarkers in epilepsy patients

**Figure 2:**
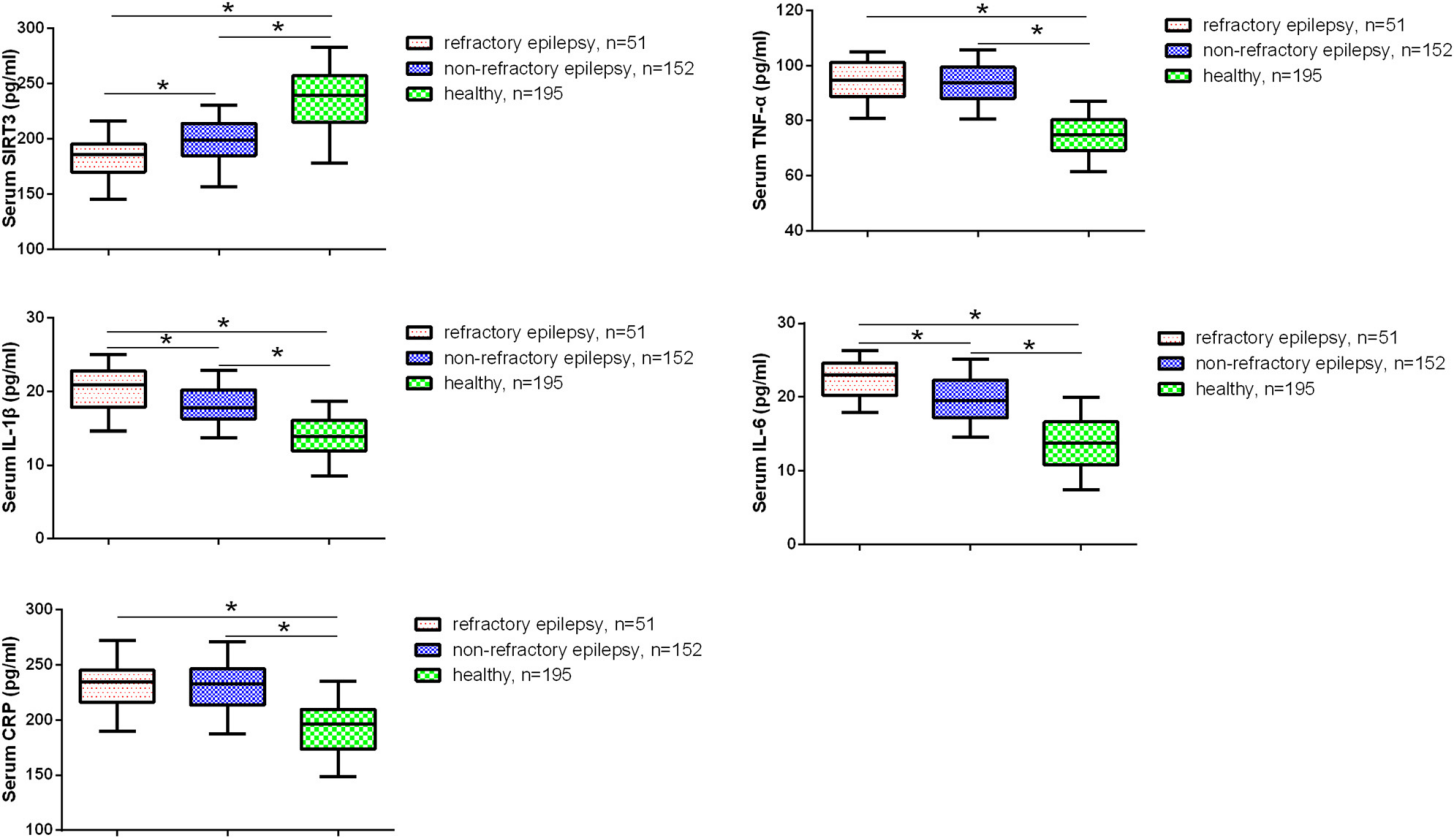
Serum cytokines levels in epilepsy patients. *P < 0.05.

Abnormal expression of certain serum biomarkers in patients with epilepsy has been confirmed in several studies. For example, elevated levels of IL-6 and IL-1β have been found in epilepsy patients [16]. Therefore, we further investigated the levels of serum biomarkers, including SIRT3, IL-6, IL-1β, TNF-α, and CRP in epilepsy patients. As shown in Figure 2 and Table 2, compared to the healthy volunteers, epilepsy patients had significantly increased serum levels of the inflammatory factors IL-6, IL-1β, TNF-α, and CRP (*P* < 0.05), while SIRT3 levels were significantly decreased. Furthermore, compared to non-refractory epilepsy patients, refractory epilepsy patients had significantly lower levels of serum SIRT3 and significantly higher levels of IL-6 and IL-1β (*P* < 0.05). No significant differences in serum TNF-α and CRP levels were found between non-refractory epilepsy patients and refractory epilepsy patients.

**Table 2:** Serum cytokines levels in all subjects

Variable	Refractory epilepsy, *n* = 51	Non-refractory epilepsy, *n* = 152	Healthy, *n* = 195	*P*1	*P*2	*P*3
SIRT3 (pg/mL)	183.16 ± 17.22	199.00 ± 18.68	228.05 ± 24.33	<0.001	<0.001	<0.001
IL-6 (pg/mL)	22.51 ± 2.53	19.70 ± 2.97	13.73 ± 3.48	<0.001	<0.001	<0.001
IL-1β (pg/mL)	20.24 ± 3.07	18.22 ± 2.46	13.93 ± 2.69	<0.001	<0.001	<0.001
TNF-α (pg/mL)	94.67 ± 6.90	93.67 ± 6.72	74.84 ± 6.64	0.627	<0.001	<0.001
CRP (pg/mL)	232.37 ± 22.15	230.23 ± 22.93	193.91 ± 23.14	0.833	<0.001	<0.001

IL: interleukin; TNF-α: tumor necrosis factor-alpha; CRP: C-reactive protein; SIRT: sirtuin. *P*1 refractory epilepsy patients compared with non-refractory epilepsy patients. *P*2 refractory epilepsy patients compared with healthy controls. *P*3 non-refractory epilepsy patients compared with healthy controls.

### 3.3 Correlations of SIRT3 with inflammatory factors and clinical characteristics

SIRT3 has been shown to be highly correlated with disease severity and prognosis in brain disorders, and animal studies have also indicated that overexpression of SIRT3 could alleviate pathological damage in epileptic rats. Therefore, we subsequently assessed the correlation between serum SIRT3 levels and levels of inflammatory biomarkers [12,17]. Subsequently, we assessed the correlations between serum SIRT3 levels and the inflammatory factor levels, as well as the clinical characteristics of the epilepsy patients using Pearson’s correlation analysis. As shown in Table 3, serum SIRT3 levels were negatively correlated with the inflammatory factors IL-6 (Pearson’s correlation −0.221, *P* = 0.002). Additionally, we found that SIRT3 levels were negatively correlated with the NHS scores (Pearson’s correlation −0.272, *P* < 0.001) of epilepsy patients, while positively correlated with MOCA scores (Pearson’s correlation 0.166, *P* = 0.018).

**Table 3:** Correlation between serum SIRT3 levels and inflammatory factors and clinical characteristics of epilepsy patients

Variable	SIRT3	
	Pearson’s correlation	*P*
IL-6	−0.221	0.002
IL-1β	−0.104	0.141
TNF-α	0.030	0.667
CRP	−0.075	0.288
MMSE	−0.025	0.723
MOCA	0.166	0.018
NHS3	−0.272	<0.001

BMI: body mass index; IL: interleukin; TNF-α: tumor necrosis factor-alpha; CRP: C-reactive protein; SIRT: sirtuin; MMSE: Mini-Mental State Examination; MOCA: Montreal Cognitive Assessment; NHS3: National Hospital Seizure Severity Scale. Pearson’s analysis was used for correlation analysis.

Moreover, all epilepsy patients were divided into two groups based on the average serum SIRT3 level of 195.02 pg/mL: the low SIRT3 group (*n* = 100) and the high SIRT3 group (*n* = 103). After comparing serum biomarkers and clinical characteristics between the two groups, we found that compared to patients in the high SIRT3 group, those in the low SIRT3 group had significantly higher NHS scores and serum IL-6 levels (Table 4). Additionally, we observed a significantly higher proportion of refractory epilepsy cases in the low SIRT3 group. These findings indicated an association between serum SIRT3 and inflammatory response, cognitive function, and the severity of epilepsy in patients.

**Table 4:** Comparison of serum biomarkers and clinical characteristics between SIRT3 high and low groups in epilepsy patients

Variable	Low SIRT3, *n* = 100	High SIRT3, *n* = 103	*P*
MMSE	23.33 ± 2.34	23.08 ± 2.39	0.452
MOCA	24.23 ± 1.84	24.67 ± 1.73	0.081
NHS	10.87 ± 2.42	9.43 ± 2.05	<0.001
IL-6 (pg/mL)	20.89 ± 3.17	19.93 ± 2.99	0.029
IL-1β (pg/mL)	18.86 ± 2.88	18.60 ± 2.65	0.497
TNF-α (pg/mL)	93.40 ± 7.13	94.42 ± 6.40	0.284
CRP (pg/mL)	232.01 ± 21.32	229.56 ± 24.00	0.443
Refractory epilepsy, *n* (%)	38 (38.0%)	13 (12.6%)	<0.001

IL: interleukin; TNF-α: tumor necrosis factor-alpha; CRP: C-reactive protein; SIRT: sirtuin; MMSE: Mini-Mental State Examination; MOCA: Montreal Cognitive Assessment; NHS3: National Hospital Seizure Severity Scale. Continuous data presented normal distribution were expressed by mean ± SD and analyzed by Student’s *t*-test. Chi square test was used for rates.

Subsequently, we performed a stratified analysis based on the NHS3 and MOCA scores of all epilepsy patients to evaluate the association between SIRT3 and the severity of the patients. As shown in Tables 5 and 6, epilepsy patients with lower NHS3 scores had significantly higher levels of SIRT3, while those with lower MOCA scores had significantly lower levels of SIRT3 (*P* < 0.05).

**Table 5:** Subgroup analysis based on NHS3 scores

Variable	Low NHS3 (<9, *n* = 70)	High NHS3 (≥9, *n* = 133)	*P*
SIRT3 (pg/mL)	199.81 ± 18.74	192.50 ± 19.55	0.011

SIRT: sirtuin; NHS3: National Hospital Seizure Severity Scale. Continuous data presented normal distribution were expressed by mean ± SD and analyzed by Student’s *t*-test.

**Table 6:** Subgroup analysis based on MOCA scores

Variable	Low MOCA (<25, *n* = 102)	High NHS3 (≥25, *n* = 101)	*P*
SIRT3 (pg/mL)	191.82 ± 18.35	198.25 ± 20.25	0.019

SIRT: sirtuin; MOCA – Montreal Cognitive Assessment; Continuous data presented normal distribution were expressed by mean ± SD and analyzed by Student’s *t*-test. Chi square test was used for rates.

### 3.4 Diagnostic value of SIRT3 in refractory epilepsy patients

**Figure 3:**
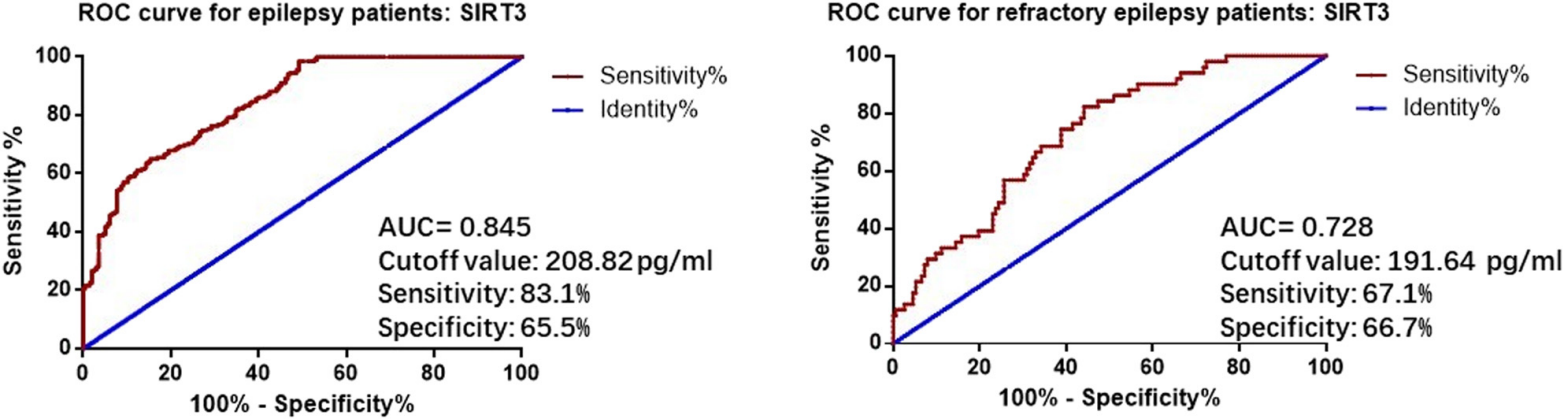
ROC curves of SIRT3 for epilepsy patients.

Furthermore, we evaluated the diagnostic value of serum SIRT3 levels in epilepsy patients using ROC curve analysis. As shown in Figure 3, the ROC curve demonstrated that serum SIRT3 could be used to diagnose epilepsy, with an area under the curve (AUC) of 0.845, a cutoff value of 208.82 pg/mL, a sensitivity of 83.1%, and a specificity of 65.5%. Moreover, we found that SIRT3 also had diagnostic value for refractory epilepsy in all epileptic patients, with an AUC of 0.728, a cutoff value of 191.64 pg/mL, a sensitivity of 67.1%, and a specificity of 66.7%.

### 3.5 Logistic regression analysis of risk factors for refractory epilepsy patients

Finally, we performed logistic regression analysis to evaluate the risk factors for refractory epilepsy patients. First, univariate logistic regression analysis was conducted, followed by multivariate logistic regression analysis with significant variables from the univariate analysis included as covariates. The results of the multivariate binary logistic regression analysis showed that SIRT3 (OR = 1.047, 95%CI = 1.027–1.067, *P* = 0.028), IL-6 (OR = 0.666, 95%CI = 0.554–0.800, *P* < 0.001), IL-1β (OR = 0.750, 95%CI = 0.630–0.894, *P* = 0.001), and NHS3 (OR = 0.555, 95%CI = 0.435–0.706, *P* < 0.001) were risk factors for refractory epilepsy (Table 7).

**Table 7:** Logistic regression of risk factors for refractory epilepsy patients

Variables	Unadjusted odds ratio	*P*	Adjusted odds ratio	*P*
IL-6	0.708 (0.627–0.807)	<0.001	0.666 (0.554–0.800)	<0.001
IL-1β	0.754 (0.664–0.857)	<0.001	0.750 (0.630–0.894)	0.001
TNF-α	0.978 (0.933–1.026)	0.361		
CRP	0.996 (0.982–1.010)	0.559		
MMSE	0.919 (0.800–1.055)	0.229		
MOCA	1.040 (0.871–1.242)	0.665		
NHS3	0.524 (0.425–0.646)	<0.001	0.555 (0.435–0.706)	<0.001
SIRT3	1.047 (1.027–1.067)	<0.001	1.028 (1.003–1.054)	0.028

BMI: body mass index; IL: interleukin; TNF-α: tumor necrosis factor-alpha; CRP: C-reactive protein; SIRT: sirtuin; MMSE: Mini-Mental State Examination; MOCA: Montreal Cognitive Assessment; NHS3: National Hospital Seizure Severity Scale.

## 4 Discussion

Although substantial progress has been made in epilepsy research, approximately 35% of epilepsy patients are resistant to AEDs and continue to experience unprovoked recurrent seizures [18]. Therefore, there is an urgent need to seek new therapeutic targets for comprehensive treatment of epilepsy patients. In this study, we found that serum SIRT3 levels were significantly decreased in epilepsy patients and were a risk factor for refractory epilepsy.

Previous studies have explored the abnormal expression of serum markers in epilepsy patients. Filimonova et al. demonstrated that the effects of brain-derived neurotrophic factor on the hippocampus and related brain structures have pro-epileptic properties [19]. Berilgen et al. found that elevated serum ghrelin may promote epileptic seizures by affecting hormone secretion and altering or disturbing physiological functions through interactions with growth hormone [20]. Liang et al.’s meta-analysis indicated that serum S100β levels were significantly elevated in epilepsy patients and serum S100B was the most valuable biomarker for epilepsy, aiding in clinical diagnosis and prognosis assessment [21]. Choi et al. confirmed that serum IL-1β levels were significantly correlated with drug resistance in pediatric epilepsy patients and were a potential prognostic biomarker for disease severity in children with epilepsy [22]. These findings are consistent with our research, as we found that serum SIRT3 and IL-6, IL-1β, TNF-α, and CRP levels were decreased significantly in epilepsy patients. The increased levels of inflammatory factors in epilepsy patients indicated the presence of neuroinflammation and highlighted the potential role of these inflammatory markers in the pathogenesis of epilepsy. These inflammatory factors may exacerbate seizure activity and influence the progression of epilepsy through specific inflammatory pathways. For example, IL-1β is primarily produced by activated microglia and astrocytes in response to central nervous system injury or inflammation [23]. IL-1β stimulation of neurons led to upregulation of p-Akt and p70S6K and promoted seizures via the PI3K/Akt/mTOR signaling pathway [24]. Inhibition of NLRP3 attenuated localized brain damage in refractory temporal lobe epilepsy by downregulating IL-1β expression [25]. Moreover, we found an association between SIRT3 and cognitive function in epilepsy patients.

Cognitive impairment often coexists with epilepsy. According to research, up to 60% of epilepsy patients report cognitive problems [26]. Seizures themselves can induce pathological damage to brain neurons, such as neuronal death or structural changes, which may directly impact cognitive function [27]. Furthermore, chronic and recurrent seizures can lead to abnormal chronic brain electrical activity, resulting in increased neuronal excitability and decreased inhibitory function. This abnormal brain electrical activity can disrupt normal information transmission and processing in the brain, thus impacting cognitive function [28,29]. SIRT3, as a mitochondrial deacetylase, may affect cognitive function by regulating mitochondrial function [30]. In recent animal studies, SIRT3 has been shown to improve cognitive dysfunction in diabetic mice by limiting the formation of abnormal mitochondrial-associated membranes [31]. Modulating SIRT3-related signaling pathways has been shown to alleviate oxidative stress and mitochondrial dysfunction, improve neuronal/synaptic injury, and ameliorate cognitive impairment [32]. In a study on AD mice, activation of the SIRT3 signaling pathway inhibited acetylation of HMGB1 induced by Aβ25-35, thereby restoring redox balance and inhibiting neuroinflammation, rescuing cognitive impairment in AD [33]. Additionally, SIRT3 has been shown to inhibit hippocampal neuroinflammation and play a critical role in the pathogenesis of postoperative cognitive dysfunction in elderly mice [34]. These findings suggested that SIRT3 might improve cognitive impairment in elderly mice by inhibiting neuroinflammation. Our findings also demonstrated that serum SIRT3 levels were negatively correlated with inflammatory factors IL-6 in epilepsy patients.

Additionally, we found that SIRT3 can be used for diagnosing epilepsy and distinguishing patients with refractory epilepsy. In other diseases, SIRT3 has been identified as a potential biomarker. For instance, in sepsis patients, SIRT3 can predict the risk of mortality [35]. In COVID-19, severely ill patients have significantly lower levels of SIRT3, and its levels are negatively correlated with CRP and procalcitonin levels, indicating the association of serum SIRT3 levels with clinical outcomes and prognosis in COVID-19 patients [36]. In intracerebral hemorrhage, the decline in SIRT3 levels is highly correlated with the severity of bleeding and poor prognosis at 90 days, suggesting that plasma SIRT3 can serve as a potential prognostic biomarker for intracerebral hemorrhage [17]. Only one animal study has focused on the mechanism of SIRT3 in epilepsy, which showed that activating SIRT3 can alleviate hippocampal pathological damage, promote mitochondrial autophagy, and reduce oxidative stress in epilepsy rats [12]. Our study added to the clinical significance of SIRT3 in epilepsy, the serum SIRT3 levels were decreased significantly in epilepsy patients and further decreased in patients with refractory epilepsy, and associated with the inflammatory status, cognitive function, and severity of epilepsy in patients and represent a risk factor for refractory epilepsy, suggesting a potential involvement of SIRT3 in the development and progression of this neurological disorder. This reduction in SIRT3 levels could have detrimental effects on mitochondrial function and oxidative stress regulation, leading to impaired energy metabolism and increased neuronal susceptibility to damage [37,38]. One possible mechanism by which SIRT3 depletion may worsen neuronal injury or seizure susceptibility is through its role in modulating mitochondria. SIRT3 is known to regulate mitochondrial biogenesis, dynamics, and function, as well as the cellular response to oxidative stress. Decreased SIRT3 levels could compromise these processes, resulting in mitochondrial dysfunction and an imbalance between reactive oxygen species production and antioxidant defense mechanisms [39,40]. Such dysregulation may further contribute to neuroinflammation, neurodegeneration, and increased seizure susceptibility.

Furthermore, understanding the underlying mechanisms by which SIRT3 influences epilepsy holds significant promise for the development of novel treatment approaches. SIRT3’s involvement in mitochondrial function, oxidative stress, and neuroinflammation suggests that modulating its activity or expression could represent a potential therapeutic target for epilepsy. A recent review has also shown that reduced SIRT3 expression or activity leads to hyperacetylation of hundreds of mitochondrial proteins, which is associated with neurological abnormalities, neuroexcitotoxicity, and neuronal cell death. Activating SIRT3 is a potential therapy for age-related brain abnormalities and neurodegenerative diseases [41]. Pharmacological interventions aimed at restoring or enhancing SIRT3 levels may help alleviate mitochondrial dysfunction, mitigate oxidative stress, and ultimately improve neuronal viability and seizure control.

There are several limitations to our study that should be acknowledged. First, this study was conducted at a single center with a relatively small sample size. The limited sample size may affect the diversity of the sample population and the generalizability of the study findings. Future studies should aim to expand the research to multiple hospitals or centers to validate the results obtained in this study. Second, our analysis only assessed a limited number of serum biomarkers, specifically IL-6, IL-1β, TNF-α, and CRP. While these markers provide valuable insights into the neuroinflammatory processes in epilepsy, there may be other important biomarkers and inflammatory pathways that were not included in our study. Another limitation is the lack of detailed assessment of potential confounding factors that could influence the relationship between SIRT3 and epilepsy. Factors such as age, gender, comorbidities, medication history, and lifestyle factors may have an impact on SIRT3 levels and epilepsy outcome. Furthermore, it is plausible that the extent of SIRT3 depletion, as well as the specific molecular pathways affected by SIRT3 dysregulation, may vary among different epilepsy subtypes, which may lead to possible limitations in the generalizability of our findings. Future studies exploring these potential variations would provide valuable insights into the complex pathophysiology of epilepsy and help identify subpopulation-specific therapeutic strategies. Lastly, further in-depth research is needed to elucidate the molecular mechanisms by which SIRT3 is involved in the development of epilepsy.

## 5 Conclusion

In conclusion, our findings demonstrated that serum SIRT3 levels were decreased significantly in epilepsy patients and further decreased in patients with refractory epilepsy. Additionally, decreased serum SIRT3 levels was one of the risk factors for refractory epilepsy. This study might provide new therapeutic targets and comprehensive treatment strategies for epilepsy patients.
